# Telomere stability and development of *ctc1* mutants are rescued by inhibition of EJ recombination pathways in a telomerase-dependent manner

**DOI:** 10.1093/nar/gku897

**Published:** 2014-10-01

**Authors:** Simon Amiard, Margaux Olivier, Elisabeth Allain, Kyuha Choi, Richard Smith-Unna, Ian R. Henderson, Charles I. White, Maria Eugenia Gallego

**Affiliations:** 1 Génétique, Reproduction et Développement, UMR CNRS 6293, Clermont Université, INSERM U1103, Aubière, France; 2 Department of Plant Sciences, University of Cambridge, Cambridge, UK

## Abstract

The telomeres of linear eukaryotic chromosomes are protected by caps consisting of evolutionarily conserved nucleoprotein complexes. Telomere dysfunction leads to recombination of chromosome ends and this can result in fusions which initiate chromosomal breakage–fusion–bridge cycles, causing genomic instability and potentially cell death or cancer. We hypothesize that in the absence of the recombination pathways implicated in these fusions, deprotected chromosome ends will instead be eroded by nucleases, also leading to the loss of genes and cell death. In this work, we set out to specifically test this hypothesis in the plant, Arabidopsis. Telomere protection in Arabidopsis implicates KU and CST and their absence leads to chromosome fusions, severe genomic instability and dramatic developmental defects. We have analysed the involvement of end-joining recombination pathways in telomere fusions and the consequences of this on genomic instability and growth. Strikingly, the absence of the multiple end-joining pathways eliminates chromosome fusion and restores normal growth and development to *cst ku80* mutant plants. It is thus the chromosomal fusions, *per se*, which are the underlying cause of the severe developmental defects. This rescue is mediated by telomerase-dependent telomere extension, revealing a competition between telomerase and end-joining recombination proteins for access to deprotected telomeres.

## INTRODUCTION

The chromosomes of most eukaryotes are linear and in consequence have two ends, which are susceptible to inappropriate recombination and degradation. Nucleoprotein structures called telomeres evolved in order to protect the ends of chromosomes and promote genomic integrity. If telomere homeostasis is impaired, they are recognized as double-strand breaks (DSBs) and become substrates for the cellular DNA Damage Repair (DDR) machinery leading to end-to-end chromosome fusions, cell cycle arrest, cell death, genome rearrangement and eventually cancer.

Although minor differences exist among species, the DNA component of telomeres is highly conserved, with telomeric DNA being composed of several kilobases of G-rich, double-stranded telomeric repeats ending with a 3′ single-strand overhang. Chromosome ends have to deal with the gradual shortening of telomeric tracts as the consequence of the unidirectionality of DNA synthesis is compensated for by telomerase, a specialized ribonucleoprotein reverse transcriptase that adds telomeric repeats at each round of replication.

Telomeric proteins are diverse. In vertebrates, telomere protection is provided mainly by shelterin, a complex of six proteins (TRF1, TRF2, POT1, TIN2, TPP1 and RAP1) that prevents inappropriate recombination and fusion between telomeres, and also has complementary roles in telomere replication and length regulation (1). TRF1 and TRF2 bind to the duplex region of the telomere through a single ‘myb-like’ DNA binding domain that contains the ‘telobox’ motif required for specific telomeric sequence recognition and POT1 binds to 3′-overhang via two OB-fold domains. No shelterin-like complex has been described in the budding yeast *Saccharomyces cerevisiae*, in which two different complexes ensure telomere protection: the Rap1–Rif1–Rif2 complex and the CST complex. Rap1 binds the double-stranded telomeric DNA and interacts with Rif1 and Rif2 which participate to the regulation of the telomere length (2). Furthermore, yKU and RAP1 are required to avoid nucleolytic telomere degradation in non-dividing cells with no role for CST complex (3). The yeast CST (Cdc13-Stn1-Ten1) complex binds the single-stranded DNA and is required for telomere protection and length regulation through the recruitment of telomerase. Yeast CST is also essential in coordinating telomere replication by promoting C-strand synthesis through a direct interaction with Pol-alpha (4) and has also been shown to be required for telomere protection specifically in S and G2/M phase (5).

The mammalian CST complex is composed of three subunits (CTC1, STN1 and TEN1) but its exact role remains elusive (4,6–7). A major difference with the yeast complex is that mammalian CST does not appear to be required for telomeric protection (8). It seems rather to be implicated in replication and particularly, replication of telomeres (9,10). In contrast to other chromosomal regions which have replication origins to both sides, telomere replication involves a single outwardly moving replication fork. This, together with the presence of G-quadruplex and T-loop structures in telomeric DNA, makes their replication potentially difficult (11). An example of the importance of this is seen in the essential role of the RTEL1 helicase for telomere replication and stability (12). In consequence, telomere replication requires additional regulation beyond that essential for general genomic replication (11,13). The mammalian CST complex is structurally related to RPA, an eukaryotic single-stranded DNA binding complex essential for general replication (4).

Only a few specific telomeric proteins have been characterized in plants (14–16) and the identification of true ‘TRF-like’ proteins in Arabidopsis is hampered by the high number of ‘myb/telobox’ domain containing proteins and their probable redundancy (17–23). Two Arabidopsis POT1 proteins (POT1a and POT1b) have been identified (24), but their roles in protection of Arabidopsis telomeres have not yet been demonstrated (25–27). The only characterized Arabidopsis proteins acting directly in telomere protection are those forming the CST complex. Mutation in any one CST subunit leads to severe morphological defects and is accompanied by massive genomic instability, telomere length decrease, single-strand G-overhang elongation, telomeric fusions and appearance of extra-chromosomal telomeric circles (7,28–29). This telomere dysfunction induces an ATR-dependent response and the CST complex thus acts to repress the ATR-dependent DDR pathway in plant cells (30,31).

Recent work has shown that the CST complex protects only half of Arabidopsis telomeres. The other half, those produced by leading-strand replication, are not subjected to resection and are blunt ended (32). The authors of this study propose that the blunt-ended extremities are protected through the binding of the KU heterodimer. In the absence of KU, these extremities would thus also be resected by EXO1 and then protected by the CST complex (32,33).

Deprotected telomeres are substrates for recombination processes leading to chromosome fusion, the ‘breakage–fusion–bridge’ cycle and cell death. In mammals, this recombination involves principally Non-Homologous End Joining (NHEJ) (34,35). The participation, or not, of the KU complex permits classification of end-joining (EJ) recombination pathways into two categories: direct joining of breaks through the KU-dependent pathway and KU-independent alternative end-joining pathways (alternative EJ) involving microhomologies (for review (36)). In Arabidopsis the KU-dependent pathway has been the subject of a number of studies (37–40). The distinction between different KU-independent pathways is not clear because both imply the use of microhomology sequences to repair the break. In vertebrates, it is known that alternative EJ is based on the action of proteins known for their roles in single-strand break repair: XRCC1, PARP1 and LIG3 (36). In Arabidopsis, the conservation of this pathway has been confirmed through studies of XRCC1 (41,42) and PARP1/PARP2 (43). Concerning the microhomology-mediated EJ pathway, the first actors identified were the MRX (MRN) and the Rad1/Rad10 (ERCC1/XPF) complexes in yeast (44). Similarly in Arabidopsis, MRE11 has been implicated in the use of microhomologies in telomere fusions (45) and XPF has been shown to be involved in a third EJ pathway of DSB repair independent of the KU complex and XRCC1 (41).

Absence of telomere capping triggers the DNA damage response and DNA repair resulting in DNA degradation, telomere fusions and chromosomal rearrangements. In the absence of recombination pathways it is assumed that ‘free’ DNA at chromosome ends will be degraded by nucleases, leading eventually to the loss of essential genes and cell death. Studies in Arabidopsis offer the opportunity to directly analyse the impact of telomere dysfunction on development and genetic stability of the organism in the absence of telomere fusions. In this study, we took advantage of the viability of multiple DNA repair mutants to analyse the involvement of the different EJ pathways in telomere fusions and the consequences of their absence in condition of non-functional CST complex.

## MATERIALS AND METHODS

### Plant material and growth conditions

The T-DNA insertion Arabidopsis mutants and PCR-based genotyping of *tert* (46), *ctc1–2* (7), *ku80* (38), *xrcc1* (42) and *xpf* (47) have been described previously.

The double mutant *ctc1 ku80* line was produced by crossing a *CTC1/ctc1* heterozygote with a *ku80* homozygote using standard techniques (see Supplementary Figure S2). The multiple mutant *ctc1 ku80 xrcc1* and *ctc1 ku80 xrcc1 xpf* lines were produced by crossing a *CTC1/ctc1 xrcc1/xrcc1* heterozygote with a *ku80 xrcc1 xpf* homozygote using standard techniques (see Supplementary Figure S5). The multiple mutant *ctc1 xrcc1* and *ctc1 xrcc1 xpf* lines were produced by crossing a *CTC1/ctc1 xrcc1/xrcc1* heterozygote with a *CTC1/ctc1 xpf/xpf* heterozygote using standard techniques (see Supplementary Figure S6). The *tert ctc1 ku80 xrcc1* and the *tert ctc1 ku80 xrcc1 xpf* lines were produced by crossing *ctc1 ku80 xrcc1* (G2) or *ctc1 ku80 xrcc1 xpf* (G2) homozygotes with a *tert ku80 xrcc1* (G2) homozygote (see Supplementary Figure S7). The double mutant *ctc1 tert* line was produced by crossing a *CTC1/ctc1* heterozygote with a *TERT/tert hetero*zygote using standard techniques (see Supplementary Figure S8).

Seeds were surface-sterilized by 7% calcium hypochlorite treatment for 15 min, rinsed four times with sterile water and sown on Petri plates on: 1x Murashige and Skoog medium including vitamins and MES buffer (#M0255; Duchefa Biochimie, Haarlem, Holland), plus 1% sucrose (Duchefa), solidified with 0.8% agar (Becton-Agar, DIFCO Laboratories, Detroit, USA). Petri dishes were placed at 4°C for 48 h and transferred to a growth chamber (16 h light, 8 h in dark), at 23°C.

### Slide preparation and immunostaining

γ-H2AX antiserum was raised and purified against a phospho-specific Arabidopsis H2AX peptide as previously described (42). For γ-H2AX immunostaining, five days after germination, root tips were prepared as described (48) with the following modifications: root tips were fixed for 45 min in 4% paraformaldehyde in solution 1xPME (50 mM Pipes, pH 6.9; 5 mM MgSO4; 1mM EGTA) and then washed 3×5 min in 1xPME. Tips were digested for 1 h in a 1% (w/v) cellulase, 0.5% (w/v) cytohelicase, 1% (w/v) pectolyase (from Sigma, St Louis, MO, USA; Refs. C1794, C8274, P5936) solution prepared in PME and then washed 3×5 min in PME. These were squashed gently onto slides, air dried and stored at −80°C.

Slides were incubated overnight at 4°C with 50 μl rabbit, anti-plant γ-H2AX antiserum diluted 1:600 (or 1:500 for anti-RPA) in fresh blocking buffer (3% BSA, 0.05% Tween-20 (Sigma) in 1x PBS), washed 3×5 min in 1x PBS solution and then incubated 2–3 h at room temperature in 50 μl blocking buffer consisting of Alexa 488-conjugated goat anti-rabbit (Molecular Probes, 1:1000) secondary antibodies. Finally, slides were washed 3×5 min in 1x PBS and mounted in Vectashield mounting medium with DAPI (40,6-diamidino-2-phenylindole) (2 μg/ml) (Vector laboratories Inc., Burlingame, CA, USA).

### DAPI staining of mitoses and Fluorescence *in situ* hybridisation

Whole inflorescences were collected, fixed and flower pistils were squashed on a slide (49). Slides were mounted using Vectashield (Vector Laboratories) mounting medium containing 1.5 μg/ml DAPI.

Fluorescence *in situ* hybridisation (FISH) was carried out according to Mokros *et al.* (50) as previously described (51), using telomeric probe labelled by PCR [(95°C 1′, 55°C 40″, 72°C 2′)*5 (94°C 1′, 60°C 40″, 72°C 2′)*25] with digoxigenin-11-dUTP using specific telomere primers 5′(TTTAGGG)3′ or Bacterial Artificial Chromosomes (BACs) from subtelomeric regions of Arabidopsis chromosomes (F6F3, F23A5, F17A22, F4P13, T20O10, F6N15, T19P19, F7J8, K9I9), labelled with biotin (Amersham) by standard nick translation reactions (Roche). For the detection of biotin-labelled probe, avidin conjugated with Texas Red (1:500, Vector Laboratories) followed by goat anti-avidin conjugated with biotin (1:100, Vector Laboratories) and avidin-Texas Red (1:500) were used. FISH after immunostaining requires a post fixation step of 30′ in 4% formaldehyde. Slides were observed by fluorescence microscopy (Zeiss Axioimager.Z1) and images were further processed and enhanced using Adobe Photoshop software.

### Microscopy and analysis of γ-H2AX

Images were acquired on the Zeiss Axioimager.Z1 microscope using Zeiss Axiovision software. Measurements were performed using the same software and images were enhanced using Adobe Photoshop software. Image stacks were captured in three dimensions (x, y, z) and were deconvolved with the deconvolution module (theoretical Point Spread Function (PSF), iterative algorithm) of Axiovision 4.6.2 (Zeiss) software to affine γ-H2AX foci, which were counted by eye. For presentation the pictures are collapsed Z-stack projections obtained using Extended-focus module (projection method) of the Axiovision 4.6.2.

### TRF analyses

Terminal restriction fragment (TRF) analysis of telomere length in Mbo1-digested genomic DNA was as previously described (52).

### Cell death assay

Seven days after germination, seedlings were immersed in propidium iodide solution (5 μg/ml in water) for 1 min and rinsed three times with water. Root tips were then transferred to slides in a drop of water and covered with a cover slip for observation under the fluorescence microscope with a Zeiss filter set 43HE (adapted from (53)).

### EdU incorporation

Arabidopsis seedlings were germinated as usual and after 7 days were transferred to liquid medium containing 10 μM of EdU for 2 h. Seedlings were then rinsed twice, transferred to fresh medium (containing 50 μM of thymidine) and fixed in 3.7% formaldehyde after 0, 6, 12 or 24 h. After permeabilization in Triton X-100 0.5%, EdU detection was performed as indicated by the manufacturer (Invitrogen Click-iT EdU Alexa Fluor 594 Imaging kit) as previously described (Amiard *et al.* (54)). Root tips were fixed for 45 min in 4% paraformaldehyde in a solution of 1 X PME (50 mM Pipes, pH 6.9, 5 mM MgSO4, 1 mM EGTA) and then washed three times for 5 min in 1X PME. Tips were digested for 1 h in a 1% (w/v) cellulase, 0.5% (w/v) cytohelicase, 1% (w/v) pectolyase (Sigma-Aldrich; Refs. C1794, C8274, P5936) solutions prepared in PME and then washed 3x5 min in PME. They were then gently squashed onto slides as described previously (Liu *et al.*, 1993), air dried and stored at −80°C.

### Genomic DNA sequencing

Somatic genomic DNA was extracted from the *ctc tert ku80 xrcc1 xpf* mutant and used to generate a sequencing library using the Tru-seq kit (Illumina). This library was subjected to paired-end sequencing on a MiSeq instrument (Illumina). The resulting reads were trimmed for adapter sequences using Trimmomatic and aligned to the TAIR10 reference sequence using Bowtie2. The resulting Sequence Alignment/Map (SAM) files were queried for mate pairs that mapped uniquely to different chromosomes. We also queried for read pairs for which at least one member mapped within 100 kb of a telomere to assay subtelomeric recombination. We used a hypergeometric test to investigate whether this was at a higher level than expected by chance, assuming recombination is uniformly distributed along the chromosomes. The mutant library was compared to a representative wild-type Col-0 library (Short Read Archive accession SRR519624) analysed in the same way as a control. A chi-square test was performed to test for significant differences in the number of chromosome–chromosome paired reads between the wild type and mutant libraries.

## RESULTS

### Absence of EJ pathways rescues the *ctc1* growth defect

The CST complex has been shown to associate with telomeres *in vivo* in Arabidopsis (7,28–29). Plants mutated for any of the three CST subunits show the loss of telomeric repeats and end-to-end chromosome fusions. Interestingly, the Riha group found that the KU complex protects the blunt-ended chromosome end while the CST complex will associate to the other chromosome end possessing the single-stranded 3′ overhang. This hypothesis was supported by the observation of high levels of chromosome end-to-end fusions in *stn1 ku80* double mutant plants. Thus deprotected telomeres in Arabidopsis are the substrates of a KU-independent non-homologous alternative EJ pathway. We have recently identified two independent alternative EJ pathways in Arabidopsis plants, dependent respectively on XRCC1 and XPF/ERCC1 complex (41). We thus asked whether deletion of both of these pathways would block telomere fusion in *ku80 ctc1* plants.

**Figure 1.**
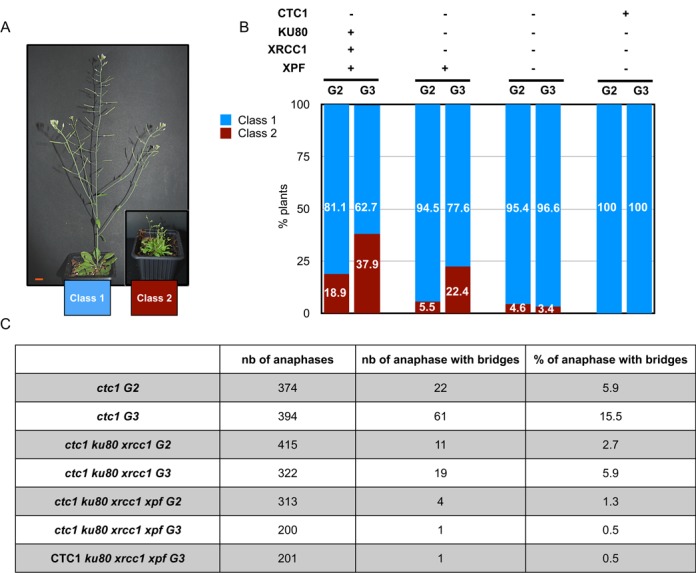
Knockout of EJ recombination rescues *ctc1* mutants. (**A**) The phenotypes of the mutants are analysed six weeks after germination. Growth phenotypes are classified as ‘wild type-like’ (class 1) or stunted, abnormal/fasciated (class 2). Bar at lower left = 1 cm. (**B**) Percentages of plants of class 1 (blue fill) and class 2 (red fill) phenotypes for second and third generation *ctc1*, *ctc1 ku80 xrcc1, ctc1 ku80 xrcc1 xpf* and *ku80 xrcc1 xpf* mutants. (**C**) Table presenting the percentage of anaphases with chromosomal bridges observed after cytogenetic analysis of flower pistil nuclei (from three different plants in each case) of *ctc1*, *ctc1 ku80 xrcc1, ctc1 ku80 xrcc1 xpf* and *ku80 xrcc1 xp*f mutants in generation 2 and 3.

Strikingly, the absence of all three EJ pathways leads to almost complete rescue of telomere-deprotected plants (Figure 1). While 18.9% of second generation (and 37.9% of third generation) of *ctc1* mutant plants are completely sterile and show severe morphological defects, only 4.6% in the second generation (and 3.4% of third generation) plants deficient for the CST complex and the three known EJ pathways show this dramatic phenotype. This improved phenotype in *ctc1 ku80 xrcc1 xpf* plants is stable at least up to the fifth generation (data not shown). The absence of increased cell cycle arrest and cell death in this quadruple mutant is in accord with this wild-type phenotype (Supplementary Figure S1). An effect is also seen in *ctc1 ku80 xrcc1* mutants, however this is not stable in subsequent generations, with 5.5% and 22.4% affected plants in second and third generations respectively (Figure 1A and B). That these effects are not simply due to the absence of the EJ pathways themselves is confirmed by the wild-type phenotype of second and third generation *ku80 xrcc1 xpf* plants.

Blocking the three EJ pathways thus very substantially restores wild-type growth and development to *ctc1* mutant plants. We thus verified whether or not this effect of blocking EJ is due to correction of the chromosome instability of *ctc1* mutants by quantifying mitotic chromosome bridges in these plants. Mitotic figures were analysed from pistils of second and third generation mutant and control plants and the results are presented in Figure 1C. As expected, we detected 5.9% and 15.5% of anaphases with bridges in second and third generation of *ctc1* mutant plants respectively. In striking contrast and in accord with their wild-type morphological phenotype, *ctc1 ku80 xrcc1 xpf* plants present almost no mitotic anaphases with bridges (0.5%), similar to *ku80 xrcc1 xpf* (0.5%). Furthermore, and in agreement with the appearance of developmental defects in the third generation, we detect 5.9% of anaphases with bridges in pistils of *ctc1 ku80 xrcc1* mutant plants.

The two alternative EJ pathways thus participate in the repair of deprotected telomeres in Arabidopsis. Absence of developmental defects in plants lacking both the CST complex and EJ proteins is thus presumably the result of the absence of chromosomal instability in these plants.

### Cells deficient for the CST and EJ proteins present long telomeric repeats

**Figure 2.**
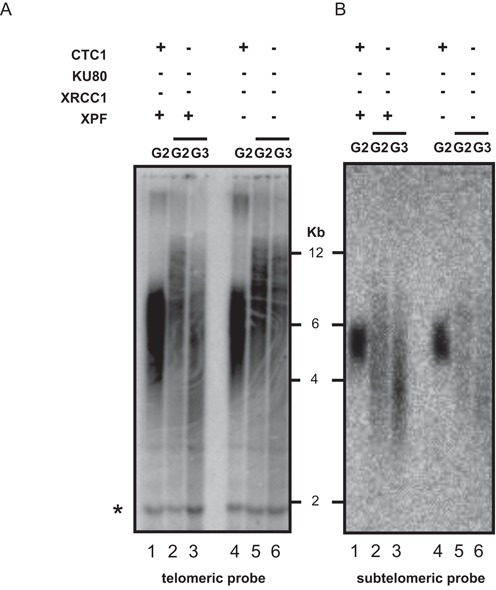
Knockout of EJ recombination induces telomere lengthening in *ctc1* mutants. TRF analysis of bulk telomere length in DNA of G2 and G3 *ctc1*, *ctc1 ku80 xrcc1* and *ctc1 ku80 xrcc1 xpf* mutants and *CTC* wild-type control plantlets using the telomeric repeat probe (**A**), and chromosome 2 subtelomeric (**B**) probes. *CTC* controls are sister plants from the same original cross.

Plants mutated for the CST complex and lacking KU80, XRCC1 and XPF are thus viable and do not present visible genomic instability. Notwithstanding, the absence of recombination at the deprotected ends of chromosomes in these plants would be expected to result in their erosion through the action of nucleases. The fact that these mutants are fertile and phenotypically wild type through at least five sexual generations however argues against this hypothesis. To clarify this we thus carried out TRF analysis to determine bulk telomere length in *ctc1 ku80* mutant plants lacking one or both alternative EJ pathways. As expected (7,38–39), we observed telomere shortening in *ctc1* mutant plants and telomere lengthening in the absence of KU, as compared to wild type (Supplementary Figure S2). Plants mutated for both the CST complex and the EJ proteins present a heterogeneous telomere length profile with some telomeres much longer than their CST wild-type siblings (Figure 2A—lanes 2 and 3 versus 1, and lanes 5 and 6 versus 4). This result was confirmed and extended by TRF analysis using a subtelomeric probe specific for the short arm of chromosome 2, which showed a wider distribution in telomere length for the *ctc1 ku80 xrcc1 xpf* mutant as related to control sibling *ku80 xrcc1 xpf* plants (Figure 2B). Further analyses showed that the generation of longer telomeres in *ctc1 ku80 xrcc1 xpf* plants is dependent upon the absence of the KU complex. Absence of XRCC1 and/or XPF in *ctc1* plants (which have the KU complex) gave similar heterogeneous telomere length profiles to those of their sibling *ctc1* plants (Supplementary Figure S3). Our data concord with the results of the Riha lab showing that, notwithstanding their very severe developmental problems, double *ctc1 ku80* mutant plants have longer telomeres as compared to *ctc1* siblings plants (Supplementary Figure S2) (32). In the absence of a functional CST complex, the absence of KU induces telomere lengthening and this is stabilized at least up to the fourth generation by mutation of the two alternative EJ pathways (Supplementary Figure S4).

### DDR in cells with deprotected telomeres in the absence of repair

**Figure 3.**
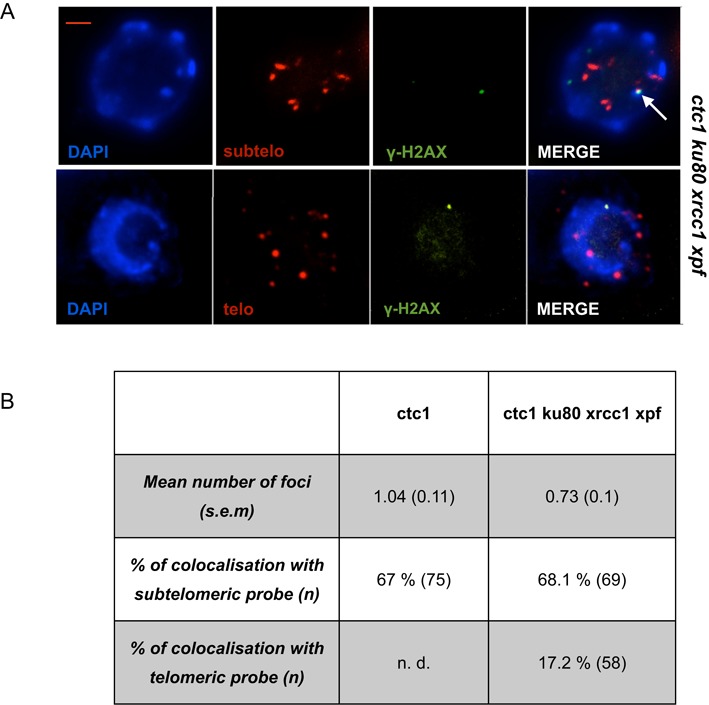
Short telomeres in *ctc1 ku80 xrcc1 xpf* mutants are deprotected. (**A**) γ-H2AX immunostaining and subtelomeric (upper panel) or telomeric (lower panel) FISH labelling of root tip nuclei of the *ctc1 ku80 xrcc1 xpf* mutants. Nuclei were stained with DAPI (blue), FISH signals are coloured in magenta and γ-H2AX foci are coloured in green. Images are collapsed Z-stack projections of a deconvolved three-dimensional image stack. The arrow (upper right image) indicates a focus with colocalized γ-H2AX and subtelomeric probe signals. Bar in (A) = 2 μm. (**B**) Table with mean number of foci per nucleus with standard error (s.e.m.), the percentages of γ-H2AX foci colocalizing with the subtelomeric and the telomeric probes. The numbers (n) of telomeric or non-telomeric foci counted are indicated in each case. n.d. means ‘not determined’.

Unprotected telomeres generated in the absence of the CST complex activate a DNA damage response, as visualized by the detection of Telomere Induced Foci (TIF), phosphorylated H2AX (γ-H2AX) foci that colocalize with telomeres (30). Given the absence of chromosomal fusions and normal growth of *ctc1* plants lacking the three EJ pathways, it is clearly of interest to determine whether or not the DDR is activated in these plants. We thus monitored the presence of γ-H2AX foci in nuclei of *ctc1 ku80 xrcc1 xpf* mutants. As shown in Figure 3A and B, γ-H2AX foci were visible in the nuclei of *ctc1* cells lacking the EJ pathway proteins, although in a slightly lower number of nuclei than the *ctc1* mutant plants (mean number of foci/nucleus of 0.73 versus 1.04 in *ctc1* single mutant). FISH was performed on the same slides using nine subtelomeric specific BACs corresponding to nine of the 10 Arabidopsis chromosome ends. As previously reported for the *ctc1* single mutant (30), 68.1% of the γ-H2AX foci colocalize with the subtelomere-specific probes revealing that *ctc1 ku80 xrcc1 xpf* telomeres are recognized as DNA damage in the mutant plants. Colocalization of γ-H2AX foci with telomeres using a probe against the telomeric repeats (TTTAGGG) however showed that only 17.2% of γ-H2AX foci colocalize with the telomeric repeat sequences. The remaining (50.9%) of TIFs defined by the subtelomeric BAC probes are thus not associated with sequences detectable by the telomeric repeat probe. The DDR is thus being induced at only a subset of dysfunctional telomeres in these plants—mostly those with telomeric sequences too short to be detected by the repeat probe. Our observations suggest that only chromosomes ends with short telomeric repeats activate a DNA damage response.

### Telomerase-dependent elongation of telomeres in *ctc1 ku80 xrcc1 xpf* mutants

**Figure 4.**
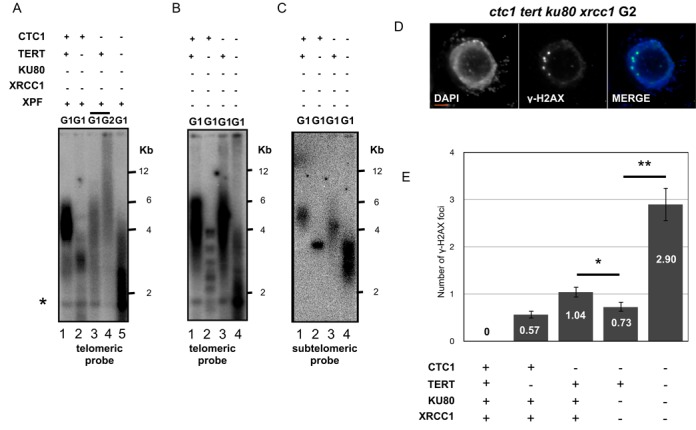
Telomere lengthening in the absence of the CST complex and EJ pathways is telomerase dependent. (**A**) TRF analysis of bulk telomere length in DNA from *ku80 xrcc1*, *tert ku80 xrcc1*, *ctc1 ku80 xrcc1*, *ctc1 tert ku80 xrcc1* plants using the telomeric repeat probe. (**B**) TRF analysis of bulk telomere length in DNA from *ku80 xrcc1 xpf*, *tert ku80 xrcc1 xpf*, *ctc1 ku80 xrcc1 xpf* and *ctc1 tert ku80 xrcc1 xpf* mutants using the telomeric repeat probe. (**C**) TRF analysis of bulk telomere length in DNA from *ku80 xrcc1 xpf*, *tert ku80 xrcc1 xpf*, *ctc1 ku80 xrcc1 xpf* and *ctc1 tert ku80 xrcc1 xpf* mutants using the chromosome 2 subtelomeric probes. (**D**) Detection of γ-H2AX immunofluorescence in mitotic root tip nuclei of second generation *ctc1 tert ku80 xrcc1* plants. DNA is stained with DAPI (blue), γ-H2AX foci are coloured in green and merged images overlay γ-H2AX foci onto chromosomes. A 2 μm scale bar is shown at the bottom left. (**E**) Mean numbers of γ-H2AX foci per nucleus in second generation wild type (WT), *tert, ctc1, ctc1 ku80 xrcc1* and *ctc1 tert ku80 xrcc1* plants. Error bars are ± s.e.m. (*n* = 100), and the asterisk indicates significant differences (Student test: *P < 0.05, **P < 0.001).

**Figure 5.**
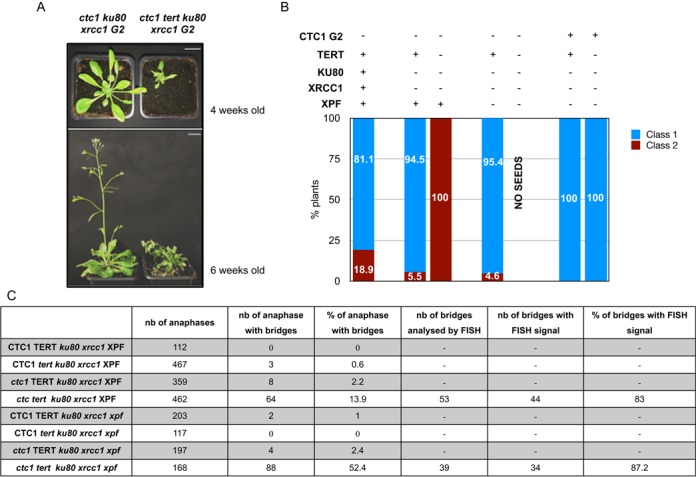
Absence of TERT worsens growth and cytogenetic defects of plants lacking CST and EJ pathway proteins. (**A**) Phenotypes of the mutants analysed four and six weeks after germination. Growth phenotypes are classified as ‘wild type-like’ (class 1) or stunted, abnormal/fasciated (class 2). Bar at lower left = 1 cm. (**B**) Percentages of plants of class 1 (blue fill) and class 2 (red fill) phenotypes for second generation *ctc1*, *ctc1 ku80 xrcc1, ctc1 tert ku80 xrcc1, ctc1 ku80 xrcc1 xpf* and *ctc1 tert ku80 xrcc1 xpf* mutants. (**C**) Table presenting the percentage of anaphases with chromosomal bridges and the percentage of anaphases with subtelomeric signal in bridges observed after cytogenetic analysis of flower pistil nuclei (from three different plants in each case) of *ku80 xrcc1, tert ku80 xrcc1, ctc1 ku80 xrcc1, ctc1 tert ku80 xrcc1, ku80 xrcc1 xpf, tert ku80 xrcc1 xpf, ctc1 ku80 xrcc1 xpf and ctc1 tert ku80 xrcc1 xpf.*

Arabidopsis *ku80*-deficient plants have longer telomeres than wild-type plants and this telomere elongation has been shown to be telomerase dependent (38,39). The new telomeric addition we observe *ctc1 ku80 xrcc1 xpf* mutant plants being dependent on the absence of KU, it seemed likely that it would be also the result of direct addition by telomerase. To test this hypothesis, we mutated the telomerase catalytic subunit (TERT) in plants lacking both the CST complex and the EJ proteins. Arabidopsis *ctc1-/- ku80 xrcc1 xpf* plants were crossed with plants mutated for *tert ku80 xrcc1* genes (see Supplementary Figure S7). Homozygous mutant lines were identified in the F2 generation and TRF analysis realized in G1 *ctc1 tert ku80 xrcc1* and *ctc1 tert ku80 xrcc1 xpf* mutant plants by Southern analysis of MboI-digested genomic DNA using the telomeric repeat probe. Results in Figure 4 clearly show a dramatic loss of telomeric repeats caused by absence of telomerase in both *ctc1 tert ku80 xrcc1 xpf* (Figure 4A) and *ctc1 tert ku80 xrcc1* (Figure 4B). The loss of repeats was faster and more heterogeneous than in sibling *CTC1* control plants (wild type for the CST complex) (lanes 5 and 2 in Figure 4A and lanes 4 and 2 in Figure 4B). This was further confirmed by examining dynamics of one particular telomere by reprobing the same Southern blot with the subtelomeric probe specific for the long arm of chromosome 2 (Figure 4C). With this probe a sharp-defined band was detected in *CTC1* plants lacking telomerase, while in the absence of the CTC1 protein a shorter and heterogeneous band was observed (lanes 4 and 2 in Figure 4C). Absence of CTC1 thus accelerates the telomere shortening of telomerase mutant plants. As expected from their loss of telomeric repeats, *ku80 xrcc1* plants lacking both CST and telomerase proteins show a 3-fold increase of γ-H2AX foci per nuclei as compared to the control plants (Figure 4D and E). This telomere shortening is accompanied by equally dramatic effects on growth, with first generation *ctc1 tert ku80 xrcc1 xpf* plants showing severe developmental defects and being completely sterile. First generation *ctc1 tert ku80 xrcc1* mutant were phenotypically wild type, however only 34% of their seeds germinated and the resulting progeny show severe developmental defects (Figure 5A and B). This contrasts clearly with second-generation plants expressing the CST complex, which present a wild-type phenotype irrespective of the presence or absence of telomerase.

We asked whether the accelerated loss of telomeric repeats in *ctc1 tert ku80 xrcc1 xpf* mutant plants was directly accompanied with an early onset of chromosomal instability. The results of the cytogenetic analysis (Figure 5C) show that while very few mitotic anaphases with bridges were detected in control plants wild type for CTC1 (with or without telomerase), 13.9% of anaphases in *ctc1 tert ku80 xrcc1 XPF* and 52.4% of the anaphases in *ctc1 tert ku80 xrcc1 xpf* mutant plants present at least one chromosome bridge. To verify the implication of chromosome ends in these fusions, we performed FISH analysis using the mixture of nine subtelomeric BAC probes. As presented in Figure 5C we found that more than 80% of anaphase bridges contain a subtelomeric FISH signal, confirming the implication of at least one chromosome end in the generation of the dicentric chromosomes.

To further analyse chromosome fusion events in *ctc tert ku80 xrcc1 xpf* mutants, we extracted genomic DNA and subjected it to paired-end Illumina sequencing. We analysed a total of ∼1 million paired-end reads and tested for incidences of mate pairs that uniquely mapped to different chromosomes. We also tested for read pairs where at least one read mapped to a subtelomere, defined as sequences within 100 kb of a telomere. In the mutant library this identified 28,748 pairs mapping to different chromosomes, of which 1735 had at least one read mapping to a subtelomere. This was significantly more than expected by chance based on a hypergeometric test, which assumes a uniform distribution of recombination along the chromosomes (*P* < 1.0×10^−15^). We compared this to a wild-type library and observed 23,808 read pairs between chromosomes, of which 449 had at least one read mapping to a subtelomere. Therefore, there was an *∼*3-fold greater incidence of chromosome–chromosome read pairs involving the subtelomeres in the mutant library, which was significant by chi-square test (*P* < 1.0×10^−15^). However, as chromosome fusions are likely to be associated with other sequence changes that will prevent read alignment we think this stringent test likely underestimates the true incidence of chromosome fusions.

Loss of telomeric repeats induced in the absence of the CST complex can thus be compensated for by the action of telomerase and this restores telomere stability, but only when the known EJ pathways are suppressed. Knocking out EJ recombination and thus telomere fusions allows telomerase access to chromosome ends in the absence of the CST complex.

### Telomerase compensates the telomere loss observed in the absence of functional CST complex

**Figure 6.**
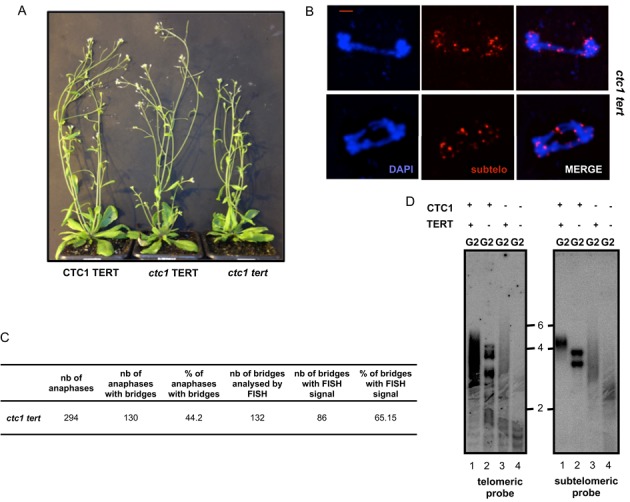
Absence of TERT in *ctc1* mutant plants leads to telomere shortening and increased cytogenetic damage. (**A**) Phenotypes of the mutants six weeks after germination. (**B**) Images of flower pistil mitotic anaphases showing chromosome bridges with subtelomeric signal in *ctc1 tert* analysed by FISH with the nine subtelomeric BAC fluorescent probes (magenta). DNA is stained with DAPI (blue). Bar = 2 μm. (**C**) Percentages of anaphases with chromosomal bridges and the percentages of anaphases with subtelomeric signal in bridges (from three different plants in each case) of *ctc1 tert.* (**D**) TRF analysis of bulk telomere length in DNA from flower buds of *CTC1 TERT, CTC1 tert, ctc1 TERT* and *ctc1 tert* mutants using telomeric (left) the chromosome 2 subtelomeric (right) probes.

We show here that the telomerase enzyme has the ability to elongate and stabilize dysfunctional *cst-* telomeres, but only in the absence of EJ recombination pathways. This suggests a competition between the telomerase and the EJ pathways for access of telomere-free ends. Considering the unexpectedly mild phenotype of early generations of the single *ctc1* mutant, we hypothesized that the telomerase is to some extent elongating telomeric ends in *ctc1* plants, even in the presence of EJ pathways. If this assumption is true, we should be able to observe a decrease in telomere length in the *ctc1 tert* double mutant. The double mutant was thus generated by crossing *tert* and *ctc1* heterozygotes. As shown in Figure 6A, *ctc1 tert* plants show a mild growth defect, but interestingly they are almost completely sterile. Only 46% (55/120) of second generation *ctc1 tert* seeds germinate and those that do result in plantlets unable to develop beyond the ‘two cotyledons’ stage. Cytogenetic analysis revealed that 44.2% of anaphases in buds of first generation *ctc1 tert* present at least one bridge and that 65.15% of bridges present a subtelomeric signal (Figure 6B and C). As expected, telomere length analyses reveal more dramatic telomere degradation in *ctc1 tert* double mutant than in either of the single mutants (Figure 6D). These results thus argue in favour of a role for telomerase in stabilizing telomeres of *ctc1* mutant plants, even in the presence of EJ recombination pathway proteins.

## DISCUSSION

In mammals, the absence of TRF2 leads to dramatic telomere fusions through the activation of Ataxia Telangiectasia Mutated (ATM) and subsequent activation of the KU-dependent EJ recombination pathway (34). In contrast, in the absence of POT1, it is the ATR kinase that is required to activate the ‘alternative’ KU-independent EJ pathway responsible for telomere fusions (35). Although Arabidopsis has two POT1 proteins, these play roles in telomerase regulation rather than end protection (25–27) and it is the absence of the CST complex which results in activation of ATR (30). We thus set out to determine which EJ pathways are responsible for telomere fusions in Arabidopsis through the combination of *cst-* and multiple recombination-knockouts.

The Arabidopsis KU complex plays an important role in telomere stability in the absence of a functional CST complex, with the absence of KU80 dramatically enhancing telomere instability in *stn1* mutants and resulting in high numbers of end-to-end chromosome fusions (32). Partly based on this data, the authors of this study proposed a novel role for KU in protection of the leading-end replicated, blunt ended half of Arabidopsis telomeres. In the absence of both CST and KU complexes, all telomeres are unprotected and this leads to elevated levels of cytogenetic and developmental defects (32,33).

The presence of high numbers of end-to-end fusions in the *stn1 ku80* plants argues for the implication of KU-independent EJ recombination pathways. Previous studies have confirmed the presence of at least two alternative KU-independent EJ pathways in Arabidopsis, implicating the protein XRCC1 and the complex ERCC1/XPF (41,42). In this study, we show that these two alternative pathways are responsible for the fusions observed in the absence of functional CST and KU complexes, with end-to-end fusions being almost completely abolished in their absence (in the *ctc1 ku80 xrcc1 xpf* mutant). Hence, the response to telomere dysfunction in the absence of CST complex is similar to that observed after POT1 deletion in mammals, with ATR-dependent signalling (30) and fusions of deprotected telomeres through alternative EJ pathways (this study).

The direct consequence of the absence of EJ pathways is the remarkable and stable improvement of the growth and development of these plants, which appear phenotypically wild type through at least five sexual generations. This concords with observations of inhibition of NHEJ in TRF2-depleted mammalian cells, in which the decrease of telomere fusions leads to markedly increased cellular survival, although no analyses were possible at the level of the ‘whole organism’ (55).

**Figure 7.**
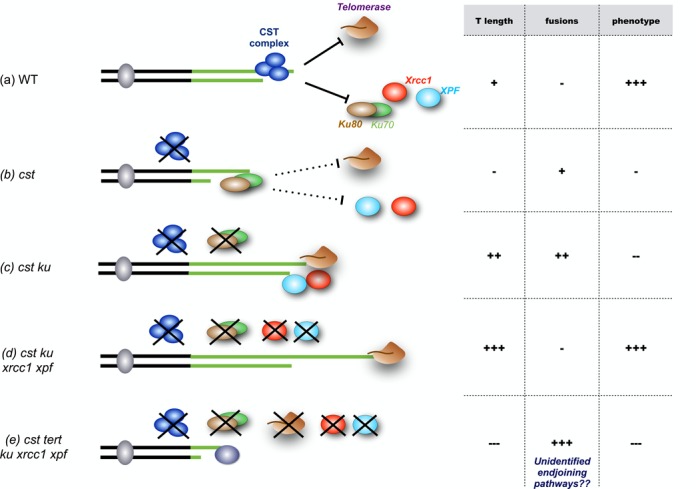
Competition between telomerase and the EJ recombination pathways at unprotected telomeres. The CST complex controls access of telomerase and EJ recombination to the chromosome ends in WT plants (**a**). In the absence of a functional CST complex (**b**), KU restricts access of telomerase to the free end and telomere shortening and fusions result in defects in growth and development. In the absence of both CST and KU (**c**), competition between alternative EJ pathways and telomerase results in both telomere lengthening and the presence of telomere fusions, accompanied by severe growth defects. Removal of these alternative EJ pathways in *cst ku80 xrcc1 xpf* mutants (**d**) opens access of telomerase to chromosome ends, extending telomeres and avoiding chromosome fusion and growth defects. Telomere loss and very high levels of chromosome fusions are seen in plants lacking CST, the EJ pathways and telomerase (**e**), and these plants show severe growth defects. See the text for details.

The principal conclusions of this work concerning the roles of CST, telomerase and EJ recombination pathways at telomeres are summarized graphically in Figure 7. Absence of EJ pathways in *ctc1* mutants not only ‘rescues’ the ability of these plants to develop normally but also results in telomere elongation. That this is due to elongation by telomerase is seen in the very severe telomere shortening and developmental defects when telomerase is absent in this genetic background: *ctc1 ku80 xrcc1 xpf* mutants appear normal, while c*tc1 ku80 xrcc1 xpf tert* mutants are very severely affected. This implies that EJ pathway proteins and telomerase compete for access of deprotected telomeres in the absence of CST. Such a competition with telomerase has already been described for the KU complex, with longer telomeres in *ku70* mutants (39), *ku80* mutants showing telomerase-dependent telomere elongation (38,39). Notwithstanding severe telomere destabilization, the absence of KU80 also gives increases in telomere length in *stn1* (32) and *ctc1* mutants (Supplementary Figure S2). We show here that XRCC1 and XPF restrict telomerase activity at telomeres in *ctc1 ku80* mutants and hypothesize that this is due to an analogous effect of competition with telomerase for access to deprotected telomeres. Deprotected *ctc1 ku80 xrcc1 xpf* telomeres are thus elongated by telomerase and no longer fuse in the absence of both classical and alternative EJ pathways, resulting in the rescue of growth and developmental phenotypes (Figure 7d). Taking away the catalytic subunit of the telomerase in this context causes rapid telomeric loss, severe genomic instability, growth defects and sterility (Figure 7e).

Given the absence of the three known EJ pathways in our *ctc1 tert ku80 xrcc1 xpf* plants, the presence of a very high level of chromosomal fusions is intriguing—do they result from unresolved replication structures or from the action of another EJ pathway? The question of the presence of uncharacterized recombination processes in Arabidopsis has already been raised by previous studies in our group. In kinetic analyses of repair after gamma irradiation of multiple recombination knockout mutants (*ku80 xrcc1 xpf xrcc2*), we observed a severe reduction in efficiency of repair. These plants were however still able to repair DSB as seen by the presence of high numbers of mitotic anaphase bridges as early as 20 min after gamma irradiation (41). This time period is far too short for cells irradiated in or before S-phase to have reached anaphase, which would argue against them simply being aberrant branched structures produced by replication. Further studies will however be needed to determine if they have a recombinational origin and if so, the specific nature of this uncharacterized ‘backup’ DSB repair pathway in response to severe genomic stress.

As mentioned above, Arabidopsis *cst* mutants activate an ATR-dependent DDR at telomeres and it is assumed that CST forms part of the plant equivalent of the shelterin complex. Surprisingly, however, first generation *ctc1* plants develop almost normally with a very low level of telomere fusions (around 2% of mitotic anaphases with fusions). *ctc1* mutants are thus able to propagate through several generations—although with increasingly severe developmental defects leading to sterility by the third or fourth generation. Such mild phenotypes contrast markedly with those of the mammalian shelterin mutants, with embryonic lethality occurring in mice depleted for TRF2 or POT1a (56,57) and deletion of TRF2 in mammalian cells leading to fusion of almost 50% of telomeres (58).

This ‘mildness’ is also seen by quantification of telomere deprotection as seen in the numbers of TIF in *cst* mutants. If CST were an essential component of the telomere capping complex it would be expected that many telomeres be unprotected in *cst* mutants. This is clearly not so in *ctc1* plants, which only show a mean of one TIF per nucleus. Furthermore, we show that TIFs in the *ctc1 ku80 xrcc1 xpf* mutant are principally localized at chromosomes without, or with very short, telomeric sequences and not to long telomeres (Figure 3). This concords with published data showing that 86% of end-to-end chromosome fusions in first generation *ctc1* mutant plants do not include telomeric repeat sequences (7). This result raises the question of the exact role of CST at telomeres in Arabidopsis. If CST were responsible for telomere protection in Arabidopsis, why do not long telomeres in *ctc1* plants become deprotected and substrates for signalling kinase and fusions?

Recent results concerning the mammalian CST complex shed some light on these questions. As in Arabidopsis, *CTC1* null Mouse embryonic fibroblasts (MEFs) exhibit γ-H2AX foci only at chromosome ends lacking telomeric repeats (8). Recent work leads to the view that the mammalian CST complex is a RPA-like complex, dedicated to help replication of particularly difficult to replicate regions of the genome such as telomeres (4). CTC1 and STN1 have been characterized as DNA polymerase-alpha accessory factors and both are required for the C-strand fill-in reaction. CST has also been shown to promote new replication origin firing and restart of stalled replication forks at telomeric and non-telomeric sites (8,9). These results thus argue for a role of the CST complex in helping the correct replication of telomere rather than in telomere protection *per se*.

In conclusion deprotected Arabidopsis telomeres fuse through the action of multiple EJ recombination pathways and it is these chromosomal fusions, *per se*, which are the underlying cause of the severe developmental and growth defects. Removal of the EJ pathways restores the growth of the plants and this is due to an opening of access of telomerase to the damaged telomeres. The EJ recombination proteins (KU80, XRCC1, XPF) thus restrict telomerase activity at deprotected telomeres.

## SUPPLEMENTARY DATA

Supplementary Data are available at NAR Online.

SUPPLEMENTARY DATA

## References

[1] Palm W., de Lange T. (2008). How shelterin protects mammalian telomeres. Annu. Rev. Genet..

[2] Wellinger R.J., Zakian V.A. (2012). Everything you ever wanted to know about Saccharomyces cerevisiae telomeres: beginning to end. Genetics.

[3] Vodenicharov M.D., Laterreur N., Wellinger R.J. (2010). Telomere capping in non-dividing yeast cells requires Yku and Rap1. EMBO J..

[4] Giraud-Panis M.-J., Teixeira M.T., Géli V., Gilson E. (2010). CST meets shelterin to keep telomeres in check. Mol. Cell.

[5] Vodenicharov M.D., Wellinger R.J. (2006). DNA degradation at unprotected telomeres in yeast is regulated by the CDK1 (Cdc28/Clb) cell-cycle kinase. Mol. Cell.

[6] Miyake Y., Nakamura M., Nabetani A., Shimamura S., Tamura M., Yonehara S., Saito M., Ishikawa F. (2009). RPA-like mammalian Ctc1-Stn1-Ten1 complex binds to single-stranded DNA and protects telomeres independently of the Pot1 pathway. Mol. Cell.

[7] Surovtseva Y.V., Churikov D., Boltz K.A., Song X., Lamb J.C., Warrington R., Leehy K., Heacock M., Price C.M., Shippen D.E. (2009). Conserved telomere maintenance component 1 interacts with STN1 and maintains chromosome ends in higher eukaryotes. Mol. Cell.

[8] Gu P., Min J.-N., Wang Y., Huang C., Peng T., Chai W., Chang S. (2012). CTC1 deletion results in defective telomere replication, leading to catastrophic telomere loss and stem cell exhaustion. EMBO J..

[9] Stewart J.A., Wang F., Chaiken M.F., Kasbek C., Chastain P.D., Wright W.E., Price C.M. (2012). Human CST promotes telomere duplex replication and general replication restart after fork stalling. EMBO J..

[10] Wang F., Stewart J.A., Kasbek C., Zhao Y., Wright W.E., Price C.M. (2012). Human CST has independent functions during telomere duplex replication and C-strand fill-in. Cell Rep..

[11] Gilson E., Géli V. (2007). How telomeres are replicated. Nat. Rev. Mol. Cell Biol..

[12] Vannier J.B., Sandhu S., Petalcorin M., Wu X., Nabi Z., Ding H., Boulton S. (2013). RTEL1 Is a replisome-associated helicase that promotes telomere and genome-wide replication. Science.

[13] Stewart J.A., Chaiken M.F., Wang F., Price C.M. (2012). Maintaining the end: roles of telomere proteins in end-protection, telomere replication and length regulation. Mutat. Res..

[14] Amiard S., Gallego M.E., White C.I. (2013). Signaling of double strand breaks and deprotected telomeres in Arabidopsis. Front. Plant Sci..

[15] Amiard S., White C., Gallego M.E. (2011). Recombination proteins and telomere stability in plants. Curr. Protein Pept. Sci..

[16] Zellinger B., Riha K. (2007). Composition of plant telomeres. Biochim. Biophys. Acta.

[17] Hwang M.G., Chung I.K., Kang B.G., Cho M.H. (2001). Sequence-specific binding property of Arabidopsis thaliana telomeric DNA binding protein 1 (AtTBP1). FEBS Lett..

[18] Karamysheva Z.N., Surovtseva Y.V., Vespa L., Shakirov E.V., Shippen D.E. (2004). A C-terminal Myb extension domain defines a novel family of double-strand telomeric DNA-binding proteins in Arabidopsis. J. Biol. Chem..

[19] Hwang M.G., Kim K., Lee W.-K., Cho M.H. (2005). AtTBP2 and AtTRP2 in Arabidopsis encode proteins that bind plant telomeric DNA and induce DNA bending in vitro. Mol. Genet. Genomics.

[20] Schrumpfová P., Kuchar M., Miková G., Skrísovská L., Kubičárová T., Fajkus J. (2004). Characterization of two Arabidopsis thaliana myb-like proteins showing affinity to telomeric DNA sequence. Genome.

[21] Peška V., Schrumpfová P.P., Fajkus J. (2011). Using the telobox to search for plant telomere binding proteins. Curr. Protein Pept. Sci..

[22] Schrumpfová P.P., Vychodilová I., Dvořáčková M., Majerská J., Dokládal L., Schořová Š., Fajkus J. (2014). Telomere Repeat Binding proteins are functional components of Arabidopsis telomeres and interact with telomerase. Plant J..

[23] Kuchar M., Fajkus J. (2004). Interactions of putative telomere-binding proteins in Arabidopsis thaliana: identification of functional TRF2 homolog in plants. FEBS Lett..

[24] Baumann P., Podell E., Cech T.R. (2002). Human Pot1 (protection of telomeres) protein: cytolocalization, gene structure, and alternative splicing. Mol. Cell. Biol..

[25] Shakirov E.V., McKnight T.D., Shippen D.E. (2009). POT1-independent single-strand telomeric DNA binding activities in Brassicaceae. Plant J..

[26] Shakirov E.V., Surovtseva Y.V., Osbun N., Shippen D.E. (2005). The Arabidopsis Pot1 and Pot2 proteins function in telomere length homeostasis and chromosome end protection. Mol. Cell. Biol..

[27] Surovtseva Y.V., Shakirov E.V., Vespa L., Osbun N., Song X., Shippen D.E. (2007). Arabidopsis POT1 associates with the telomerase RNP and is required for telomere maintenance. EMBO J..

[28] Song X., Leehy K., Warrington R.T., Lamb J.C., Surovtseva Y.V., Shippen D.E. (2008). STN1 protects chromosome ends in Arabidopsis thaliana. Proc. Natl Acad. Sci. U.S.A..

[29] Leehy K.A., Lee J.R., Song X., Renfrew K.B., Shippen D.E. (2013). MERISTEM DISORGANIZATION1 encodes TEN1, an essential telomere protein that modulates telomerase processivity in Arabidopsis. Plant Cell.

[30] Amiard S., Depeiges A., Allain E., White C.I., Gallego M.E. (2011). Arabidopsis ATM and ATR kinases prevent propagation of genome damage caused by telomere dysfunction. Plant Cell.

[31] Boltz K.A., Leehy K., Song X., Nelson A.D., Shippen D.E. (2012). ATR cooperates with CTC1 and STN1 to maintain telomeres and genome integrity in Arabidopsis. Mol. Biol. Cell.

[32] Kazda A., Zellinger B., Rössler M., Derboven E., Kusenda B., Riha K. (2012). Chromosome end protection by blunt-ended telomeres. Genes Dev..

[33] Nelson A.D.L., Shippen D.E. (2012). Blunt-ended telomeres: an alternative ending to the replication and end protection stories. Genes Dev..

[34] Smogorzewska A., Karlseder J., Holtgreve-Grez H., Jauch A., de Lange T. (2002). DNA ligase IV-dependent NHEJ of deprotected mammalian telomeres in G1 and G2. Curr. Biol..

[35] Rai R., Zheng H., He H., Luo Y., Multani A., Carpenter P.B., Chang S. (2010). The function of classical and alternative non-homologous end-joining pathways in the fusion of dysfunctional telomeres. EMBO J..

[36] Decottignies A. (2013). Alternative end-joining mechanisms: a historical perspective. Front. Genet..

[37] Friesner J., Britt A.B. (2003). Ku80- and DNA ligase IV-deficient plants are sensitive to ionizing radiation and defective in T-DNA integration. Plant J..

[38] Gallego M.E., Jalut N., White C.I. (2003). Telomerase dependence of telomere lengthening in Ku80 mutant Arabidopsis. Plant Cell.

[39] Riha K., Watson J.M., Parkey J., Shippen D.E. (2002). Telomere length deregulation and enhanced sensitivity to genotoxic stress in Arabidopsis mutants deficient in Ku70. EMBO J..

[40] van Attikum H., Bundock P., Overmeer R.M., Lee L.-Y., Gelvin S.B., Hooykaas P.J.J. (2003). The Arabidopsis AtLIG4 gene is required for the repair of DNA damage, but not for the integration of Agrobacterium T-DNA. Nucleic Acids Res..

[41] Charbonnel C., Allain E., Gallego M.E., White C.I. (2011). Kinetic analysis of DNA double-strand break repair pathways in Arabidopsis. DNA Repair.

[42] Charbonnel C., Gallego M.E., White C.I. (2010). Xrcc1-dependent and Ku-dependent DNA double-strand break repair kinetics in Arabidopsis plants. Plant J..

[43] Jia Q., den Dulk-Ras A., Shen H., Hooykaas P.J., de Pater S. (2013). Poly(ADP-ribose)polymerases are involved in microhomology mediated back-up non-homologous end joining in Arabidopsis thaliana. Plant Mol. Biol..

[44] Ma J.-L., Kim E.M., Haber J.E., Lee S.E. (2003). Yeast Mre11 and Rad1 proteins define a Ku-independent mechanism to repair double-strand breaks lacking overlapping end sequences. Mol. Cell. Biol..

[45] Heacock M., Spangler E., Riha K., Puizina J., Shippen D.E. (2004). Molecular analysis of telomere fusions in Arabidopsis: multiple pathways for chromosome end-joining. EMBO J..

[46] Fitzgerald M.S., Riha K., Gao F., Ren S., McKnight T.D., Shippen D.E. (1999). Disruption of the telomerase catalytic subunit gene from Arabidopsis inactivates telomerase and leads to a slow loss of telomeric DNA. Proc. Natl Acad. Sci. U.S.A..

[47] Dubest S., Gallego M.E., White C.I. (2002). Role of the AtRad1p endonuclease in homologous recombination in plants. EMBO Rep..

[48] Friesner J.D., Liu B., Culligan K., Britt A.B. (2005). Ionizing radiation-dependent gamma-H2AX focus formation requires ataxia telangiectasia mutated and ataxia telangiectasia mutated and Rad3-related. Mol. Biol. Cell.

[49] Caryl A.P., Armstrong S.J., Jones G.H., Franklin F.C. (2000). A homologue of the yeast HOP1 gene is inactivated in the Arabidopsis meiotic mutant asy1. Chromosoma.

[50] Mokros P., Vrbsky J., Siroky J. (2006). Identification of chromosomal fusion sites in Arabidopsis mutants using sequential bicolour BAC-FISH. Genome.

[51] Vannier J.-B., Depeiges A., White C., Gallego M.E., Grelon M. (2009). ERCC1/XPF protects short telomeres from homologous recombination in Arabidopsis thaliana. PLoS Genet..

[52] Gallego M.E., White C.I. (2001). RAD50 function is essential for telomere maintenance in Arabidopsis. Proc. Natl Acad. Sci. U.S.A..

[53] Curtis M.J., Hays J.B. (2007). Tolerance of dividing cells to replication stress in UVB-irradiated Arabidopsis roots: requirements for DNA translesion polymerases eta and zeta. DNA Repair.

[54] Amiard S., Charbonnel C., Allain E., Depeiges A., White C.I., Gallego M.E. (2010). Distinct roles of the ATR kinase and the Mre11-Rad50-Nbs1 complex in the maintenance of chromosomal stability in Arabidopsis. Plant cell.

[55] Peuscher M.H., Jacobs J.J.L. (2011). DNA-damage response and repair activities at uncapped telomeres depend on RNF8. Nat. Cell Biol..

[56] Celli G.B., De Lange T. (2005). DNA processing is not required for ATM-mediated telomere damage response after TRF2 deletion. Nat. Cell Biol..

[57] Hockemeyer D., Daniels J., Takai H., Delange T. (2006). Recent expansion of the telomeric complex in rodents: two distinct pot1 proteins protect mouse telomeres. Cell.

[58] Takai H., Smogorzewska A., de Lange T. (2003). DNA damage foci at dysfunctional telomeres. Curr. Biol..

