# Supplementary material for: Telomere stability and development of ctc1 mutants are rescued by inhibition of EJ recombination pathways in a telomerase-dependent manner
Source: Nucleic Acids Res. 2014 Oct 1;42(19):11979–91. doi: 10.1093/nar/gku897 (PMC4231758; doi:10.1093/nar/gku897)
Supplement: SUPPLEMENTARY DATA [file supp_42_19_11979__index.html]

Telomere stability and development of ctc1 mutants are rescued by inhibition of EJ recombination pathways in a telomerase-dependent manner — Telomere stability and development of ctc1 mutants are rescued by inhibition of EJ recombination pathways in a telomerase-dependent manner — SUPPLEMENTARY DATA 

# Telomere stability and development of *ctc1* mutants are rescued by inhibition of EJ recombination pathways in a telomerase-dependent manner

## SUPPLEMENTARY DATA

**Files in this Data Supplement:**

- SUPPLEMENTARY DATA
