# Supplementary material for: Telomere stability and development of ctc1 mutants are rescued by inhibition of EJ recombination pathways in a telomerase-dependent manner
Source: Nucleic Acids Res. 2014 Oct 1;42(19):11979–91. doi: 10.1093/nar/gku897 (PMC4231758; doi:10.1093/nar/gku897)
Supplement: SUPPLEMENTARY DATA [file supp_gku897_nar-00687-x-2014-File009.pdf]

Amiard_SuplFig1

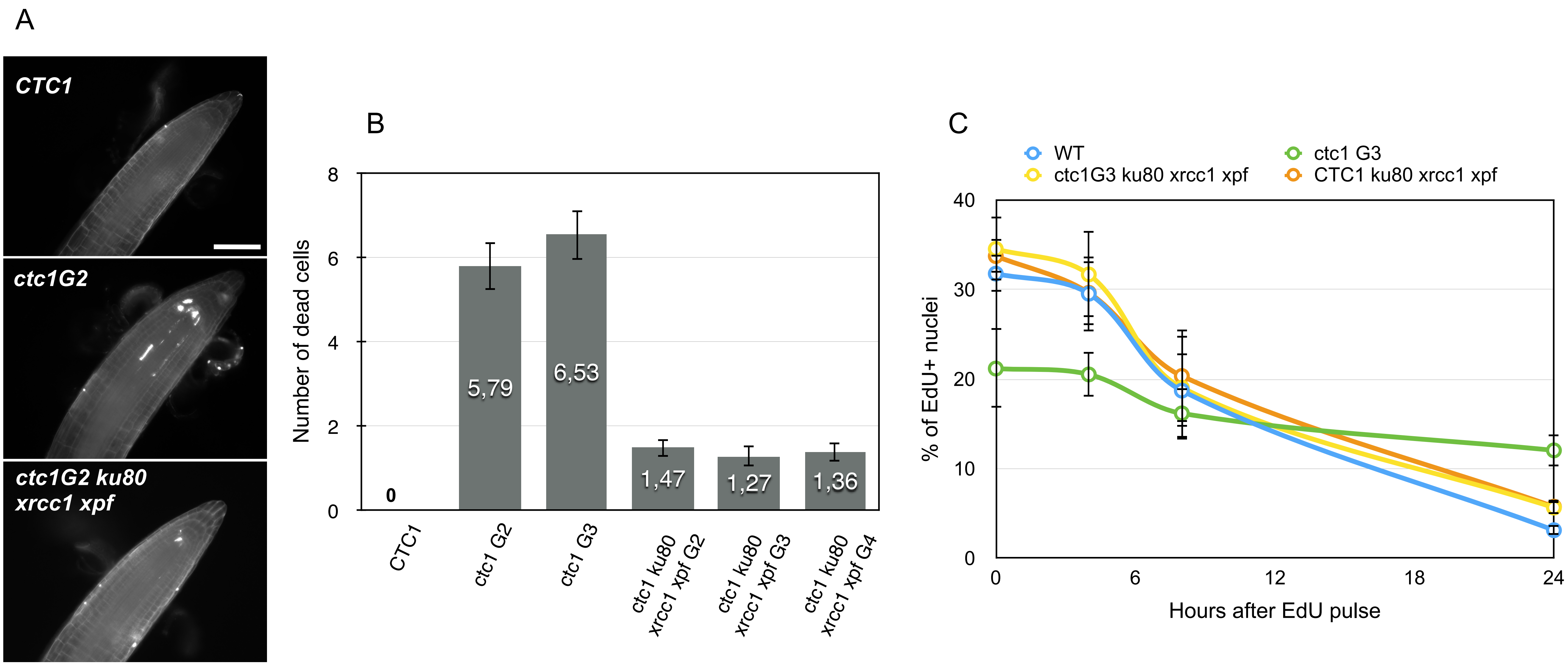

Supplemental Figure 1:

(A) Representative images of root tips with dead cells stained with Propidium Iodide. No cell death is observed in WT (CTC1. upper image). While abundant cell death is observed in the region around the quiescent center in second generation (G2) *ctc1* plants (middle), this is substantially alleviated by the absence of EJ recombination pathways in G2 *ctc1 ku80 xrcc1 xpf* plants (lower). (B) Quantification of numbers of dead cells per root tip confirms this observation and shows that the absence of EJ recombination pathways in *ctc1 ku80 xrcc1 xpf* plants stably reduces cell death up to at least generation 4. Mean numbers of dead cells per root tip and standard errors are given from counts of at least 10 root tips in each case. (C) Cell division slow-down in mitotic root cells of third generation ctc1 mutant plants is rescued by removing the EJ pathways (ctc1 G3 ku80 xrcc1 xpf), of EdU Pulse-chase experiment showing loss of EdU labelling 0, 4, 8 or 24h after a 1h EdU pulse (greater than 1000 nuclei counted in each case).

A

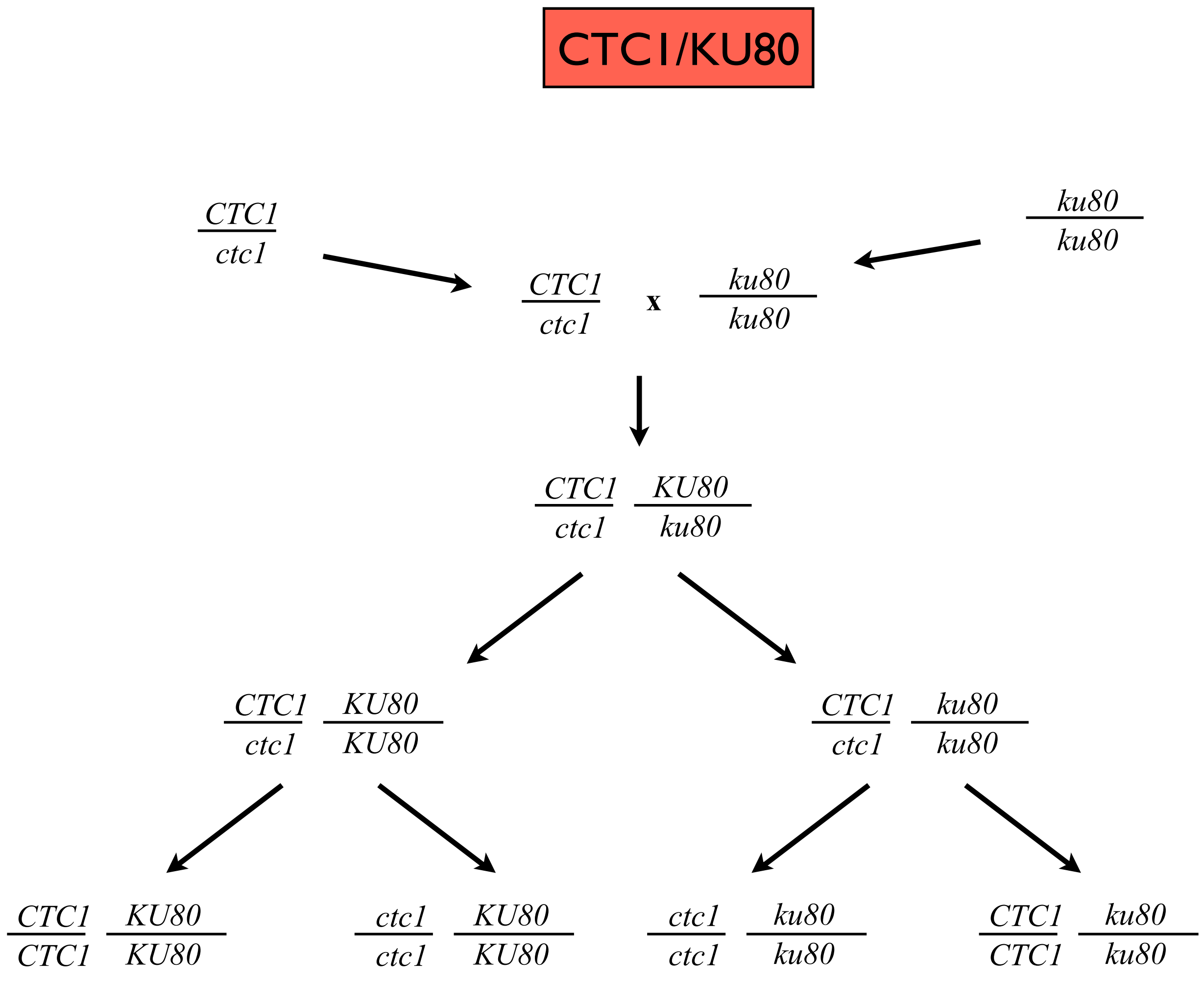

B

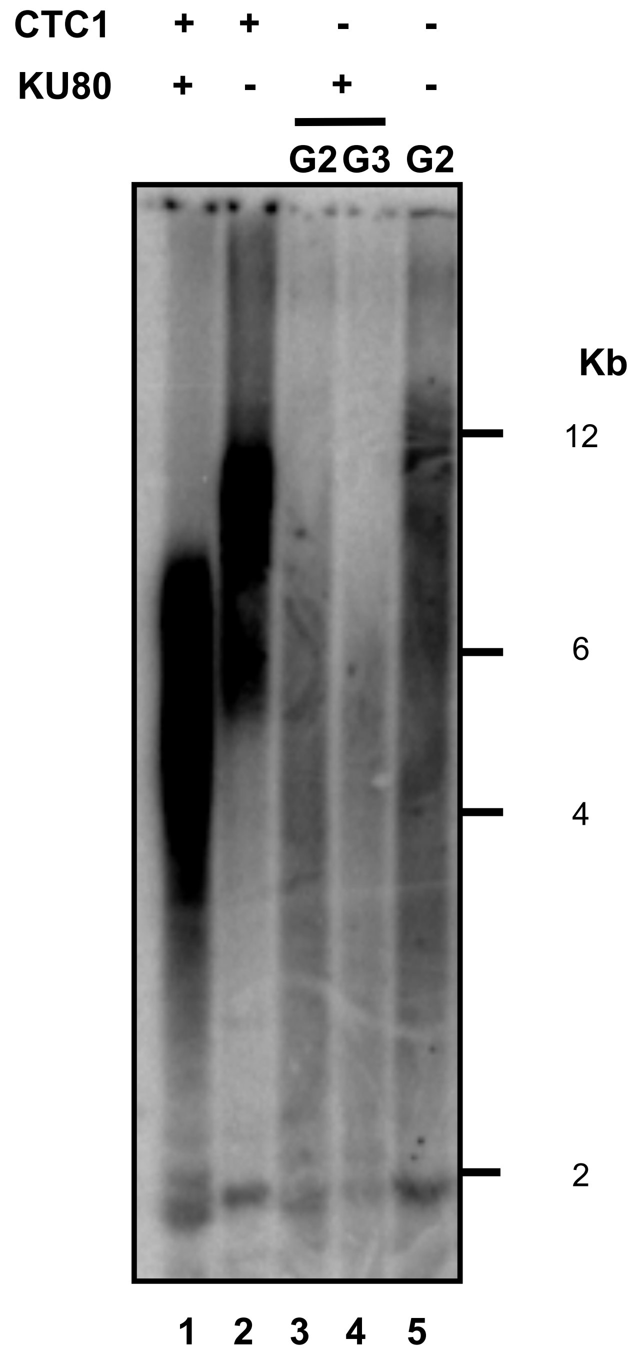

Supplemental Figure 2:

A) Schema detailing the cross between CTC1 and KU80.

B)TRF analysis of bulk telomere length in DNA from flower buds of *WT, ku80, ctc1* and *ctc1 ku80 mutants.* Telomere repeat probe.

Amiard_SuplFig3

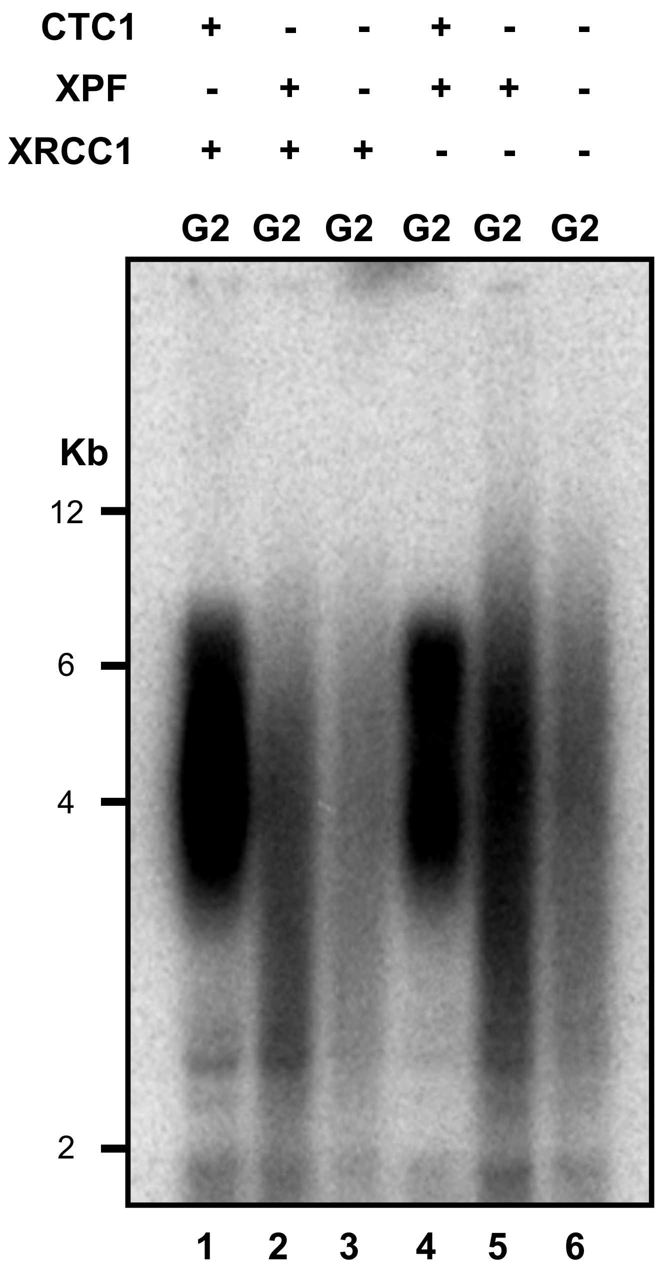

Supplemental Figure 3:

Absence of XRCC1 and/or XPF in *ctc1* plants gives similar heterogeneous telomere length profiles as their sibling *ctc1* plants. TRF analysis of bulk telomere length in DNA from flower buds of *xpf, xrcc1, ctc1, ctc1 xpf, ctc1 xrcc1* and *ctc1 xpf xrcc1 mutants.* Telomere repeat probe.

Amiard_SuplFig4

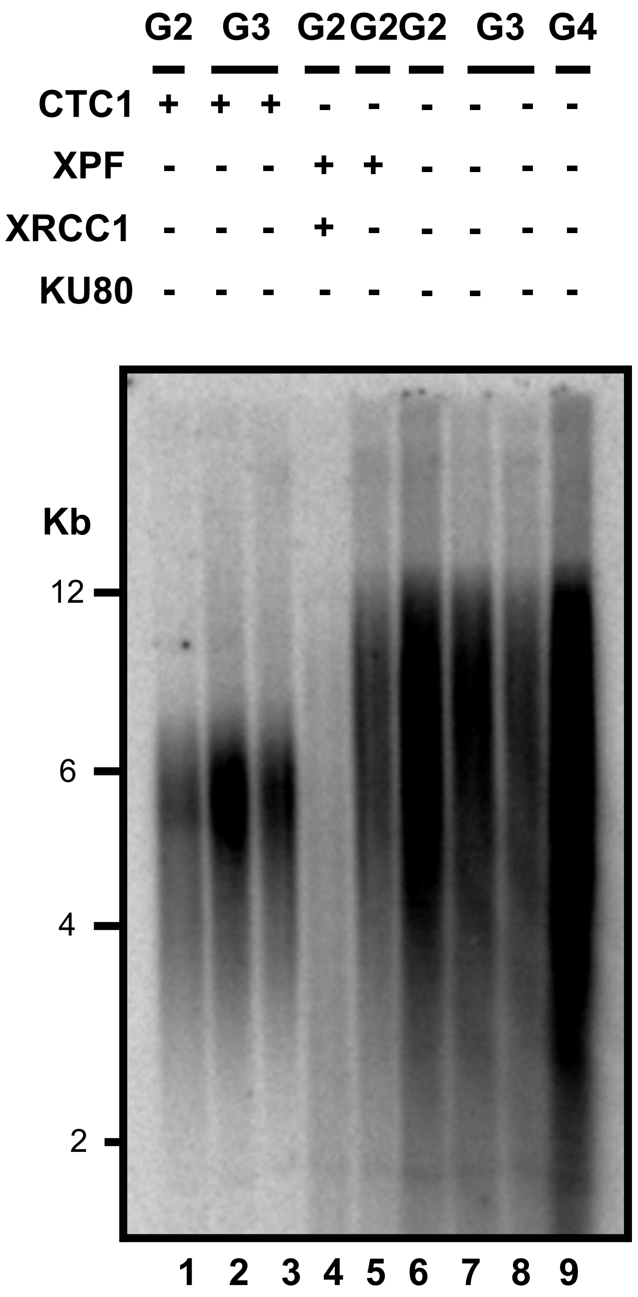

Supplemental Figure 4: absence of KU induces telomeres lengthening.

The absence of KU in *ctc1* mutants induces telomere lengthening and this is stabilised at least up to the fourth generation by mutation of the two alternative EJ pathways. TRF analysis of bulk telomere length in DNA from flower buds of *ku80 xrcc1 xpf*, *ctc1 ku80, ctc1 ku80 xrcc1* and *ctc1 ku80 xrcc1 xpf* mutants at different generations. Telomere repeat probe.

Amiard_SuplFig5

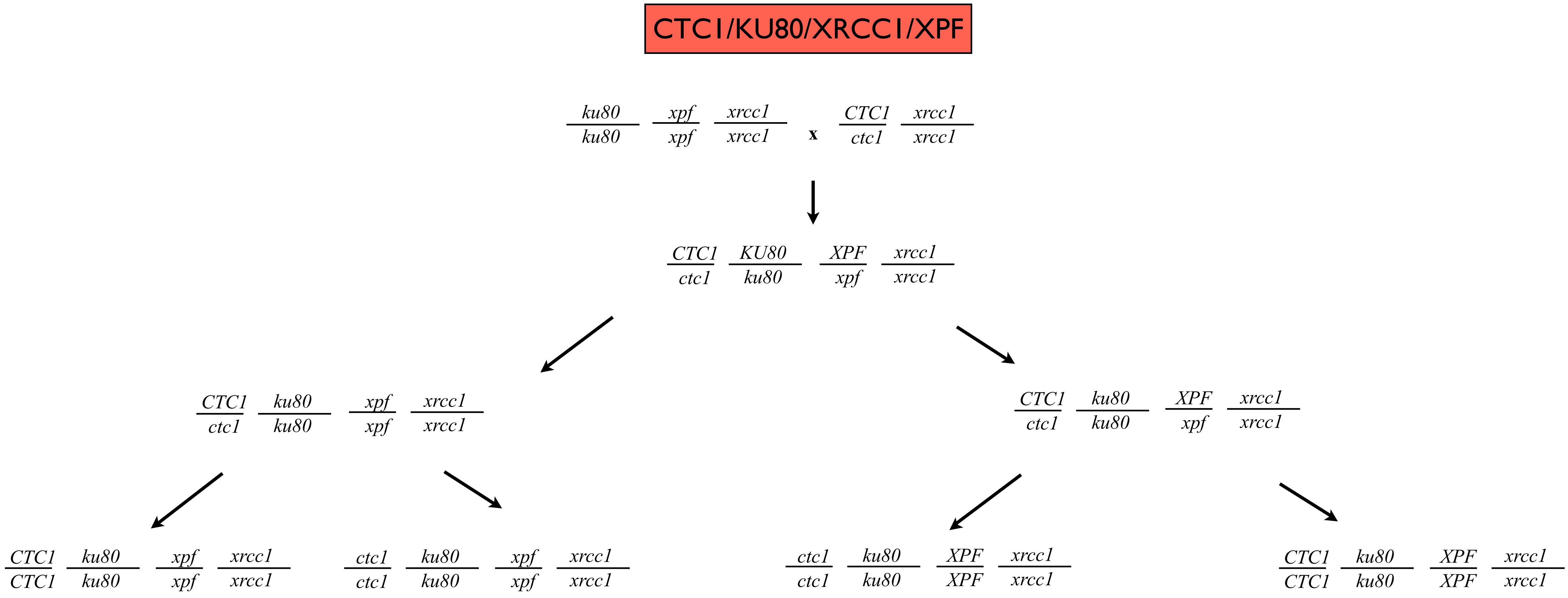

Supplemental Figure 5:

Schema detailing the cross between *ku80 xpf xrcc1* and CTC1/*ctc1 xrcc1* plants.

Amiard_SuplFig6

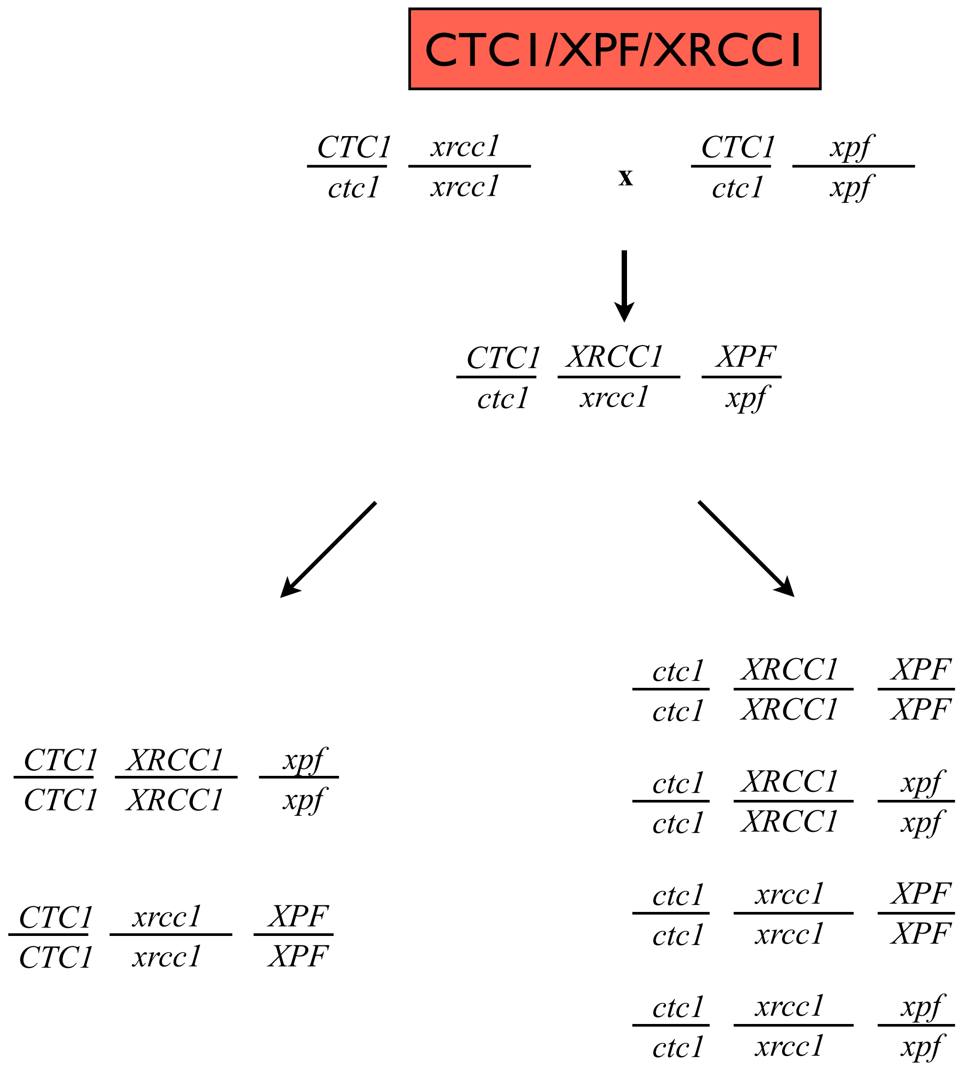

Supplemental Figure 6:

Schema detailing the cross between *CTC1/ctc1 xrcc1* and *CTC1/ctc1 xpf* plants.

Amiard_SuplFig7

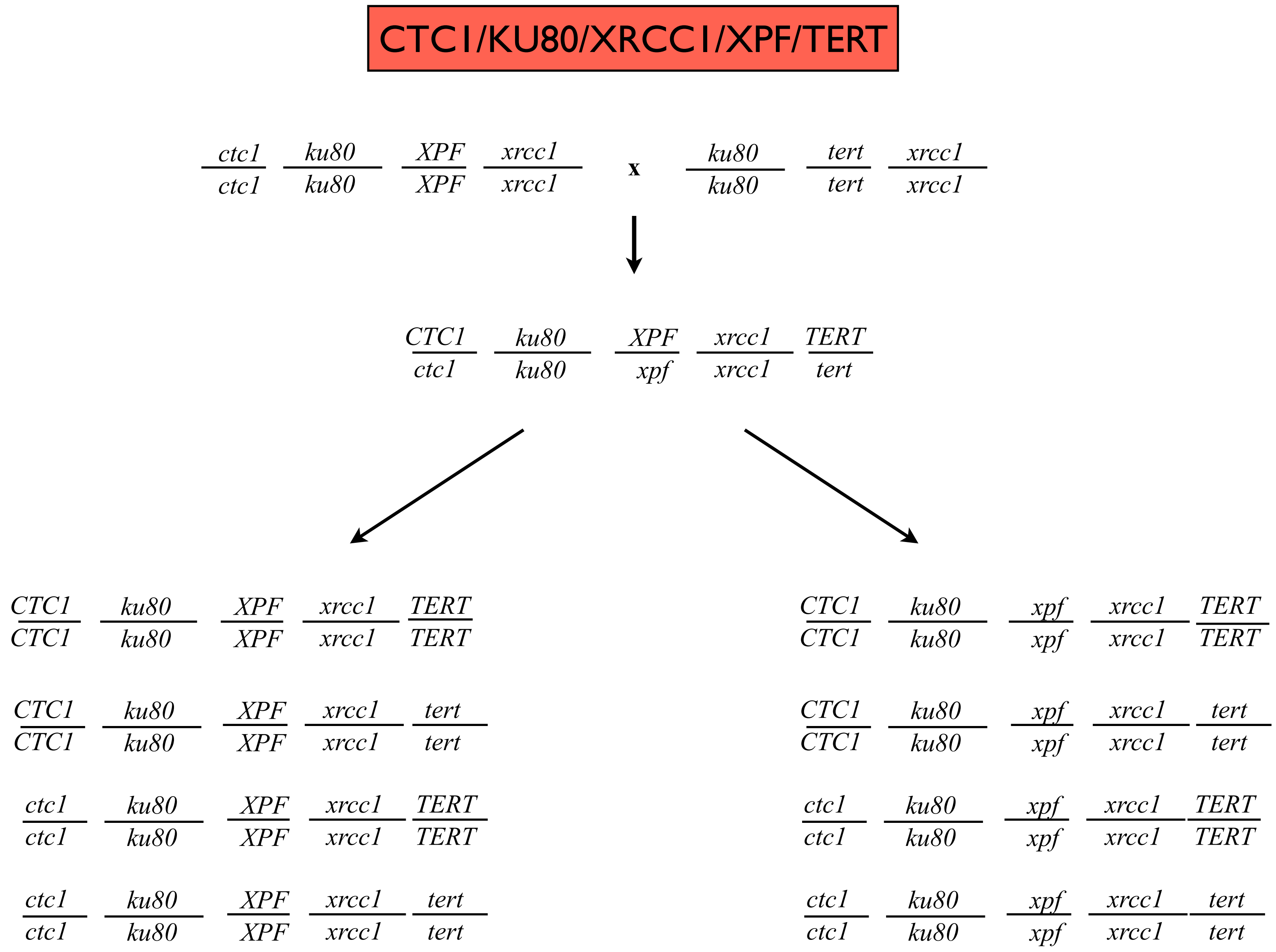

Supplemental Figure 7:

Schema detailing the cross between *ctc1 ku80 xrcc1* (G2) and *ku80 tert xrcc1* (G2) plants.

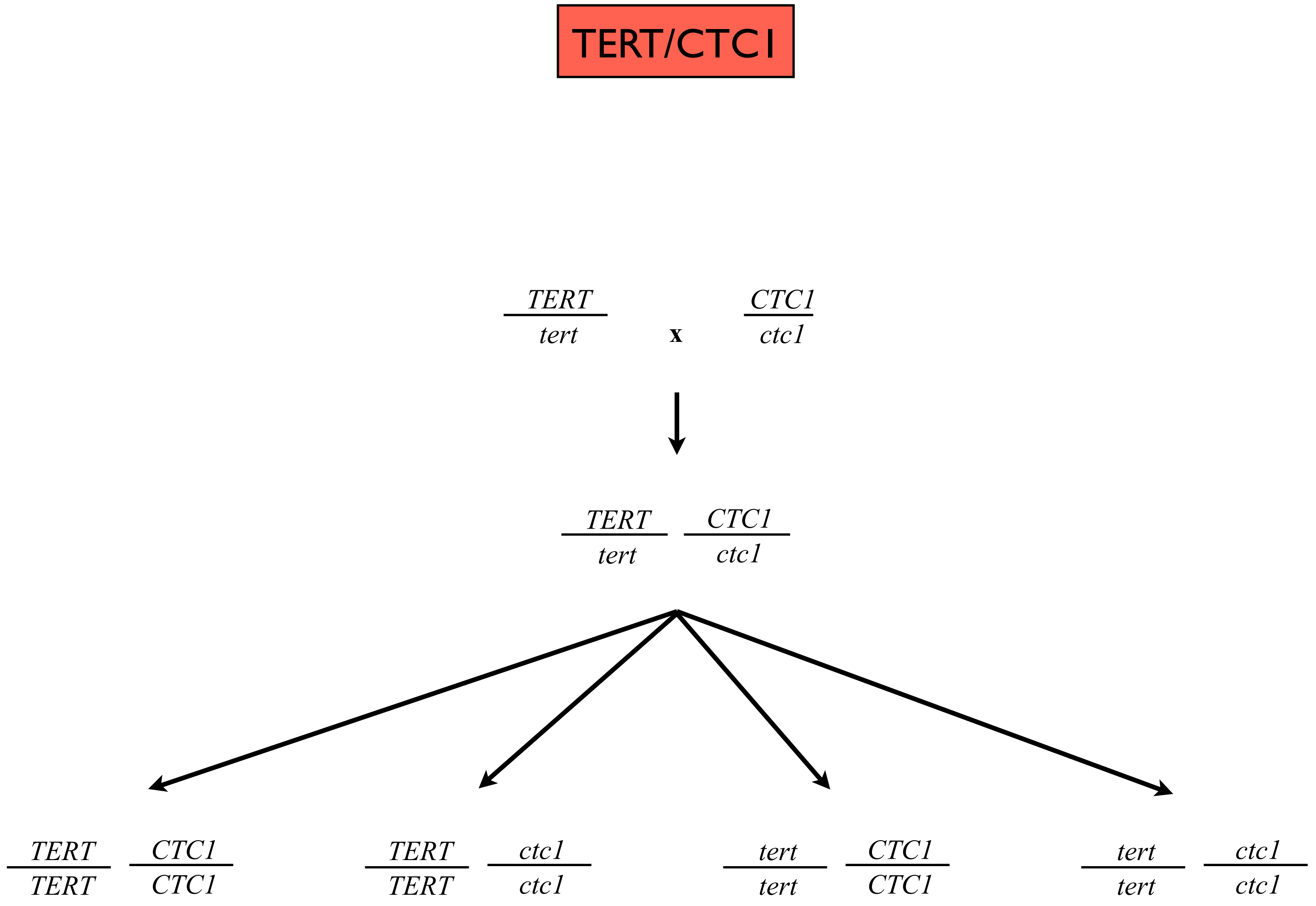

Supplemental Figure 8:

Schema detailing the cross between *TERT/tert* and *CTC1/ctc1* plants.
